# Effect of an integrated maternal and neonatal health intervention on maternal healthcare utilisation addressing inequity in Rural Bangladesh

**DOI:** 10.1186/s13690-023-01155-7

**Published:** 2023-08-22

**Authors:** Anisuddin Ahmed, Fariya Rahman, Abu Sayeed, Tania Sultana Tanwi, Abu Bakkar Siddique, Aniqa Tasnim Hossain, Saraban Tahura Ether, Ema Akter, Tazeen Tahsina, Syed Moshfiqur Rahman, Shams El Arifeen, Ahmed Ehsanur Rahman

**Affiliations:** 1 Department of Women’s and Children’s Health, Uppsala University, Uppsala, 75205 Sweden; 2 International Centre for Diarrheal Disease Research, Bangladesh, Shaheed Tajuddin Ahmed Sarani, 1212 Mohakhali, Dhaka Bangladesh

**Keywords:** Maternal health, Neonatal Health, Skilled ANC visit, 4+ ANC check-up, Skilled delivery, Skilled PNC, Skilled Healthcare Provider, Bangladesh

## Abstract

**Background:**

Although Bangladesh has made significant improvements in maternal, neonatal, and child health, the disparity between rich and poor remains a matter of concern.

**Objective:**

The study aimed to increase coverage of skilled maternal healthcare services while minimising the inequity gap among mothers in different socioeconomic groups.

**Methods:**

We implemented an integrated maternal and neonatal health (MNH) intervention between 2009 and 2012, in Shahjadpur sub-district of Sirajganj district, Bangladesh. The study was quasi-experimental in design for the evaluation. Socioeconomic status was derived from household assets using principal component analysis. Inequity in maternal healthcare utilisation was calculated using rich-poor ratio and concentration index to determine the changes in inequity between the baseline and the endline time period.

**Result:**

The baseline and endline surveys included 3,158 (mean age 23.5 years) and 3,540 (mean age 24.3 years) recently delivered mothers respectively. Reduction in the rich-poor ratio was observed in the utilisation of skilled 4+ antenatal care (ANC) (2.4:1 to 1.1:1) and related concentration index decreased from 0.220 to 0.013 (p < 0.001). The rich-poor ratio for skilled childbirth reduced from 1.7:1 to 1.0:1 and the related concentration index declined from 0.161 to -0.021 (p < 0.001). A similar reduction was also observed in the utilisation of skilled postnatal care (PNC); where the rich-poor gap decreased from 2.5:1 to 1.0:1 and the related concentration index declined from 0.197 to -0.004 (p < 0.001).

**Conclusion:**

The MNH intervention was successful in reducing inequity in receiving skilled 4+ ANC, delivery, and PNC in rural Bangladesh.

**Table:** 

**Text box 1. Contributions to the literature**
• Socioeconomic disparity in availing essential MNCH services is a major challenge globally and nationally in Bangladesh.
• The intervention tested substantially contributed to reducing the rich-poor gaps in accessing skilled maternal healthcare services such as antepartum, intrapartum, and postpartum care from medically trained providers.
• Strengthening the health system through refresher training on MNCH services to the skilled health workforce and creating community awareness of MNCH services by involving community people would help in reducing the existing inequity and achieving the universal health coverage of SDG target 3 for Bangladesh.

## Introduction

Universal Health Coverage (UHC) is a global agenda aiming to ensure health services at a reasonable cost as per individual requirements [1]. Three Maternal, Neonatal, and Child Health (MNCH) services covered under a UHC package are antenatal care (ANC), skilled delivery, and postnatal care (PNC) [2]. These are considered effective means to mitigate pregnancy, delivery, and postnatal complications and thus are beneficial to reduce maternal and neonatal mortality [3–5]. Despite having a UHC scheme, many countries around the world are still struggling to bridge the MNCH service gap between rich and poor communities [6]. In 2015, the State of Inequity report comprised data on MNCH inequity-prone low- and middle- income countries [6]. Among the 86 countries, the report showed that the richest women benefit from skilled delivery more than the poorest women by more than 80% and almost half of the countries showed a 25% difference among the richest and the poorest women in ANC uptake [6]. Therefore, the global drive to reduce maternal mortality and other adverse maternal health outcomes is not inclusive of achieving equity. It is important to ensure access to essential maternal, neonatal, and child healthcare services, irrespective of household socioeconomic status in order to fully achieve the Sustainable Development Goal (SDG) targets [7].

With persisting socioeconomic differences, the inequity scenario in Bangladesh is similar to this global finding. Bangladesh Demographic and Health Survey (BDHS) 2017-18 measured that the skilled ANC seeking between the richest and the poorest quintile differs by 34% (97.2% vs. 63.6%) (8). Likewise, the health facility delivery is higher among the women of the highest wealth quintile in comparison to the lowest wealth quintile (77.9% vs. 26.3%) and a similar rich-poor gap is also observed in PNC coverage as well (8). According to BDHS 2017-18, almost 71.5% of the mothers from the lowest wealth quintile do not avail PNC services at all but for the highest wealth quintile this figure is 17.9% (8).

To act upon service gaps and improve MNCH status, several governmental and non-governmental programmes have operated in Bangladesh since 2000. Programmes include the Maternal Health Voucher Scheme (MHVS), Emergency Obstetrical Care Services (EmOC) and Government’s nationwide training programmes for community skilled birth attendants (CSBAs) and midwives, in order to increase coverage of skilled healthcare provider’s care during pregnancy and childbirth [9, 10]. However, insufficient implementation and progress of these efforts might contribute to the continuing high maternal mortality of the country [11, 12].

Hence, an integrated evidence-based maternal and neonatal (MNH) intervention package was implemented by the International Centre of Diarrheal Disease Research, Bangladesh (icddr, b) in partnership with the Bangladesh Government spanning 4 years in a rural sub-district, with the aim to strengthen the existing healthcare system. The package contained birth and newborn care preparedness counselling, updated safe delivery-kit, management of postpartum haemorrhage (PPH) through routine implementation of active management of third stage labor (AMTSL), misoprostol and safe blood transfusion, management of eclampsia by MgSO_4_, home-based essential newborn care by CSBAs, formation of community support groups (CSGs) for community sensitisation as well as sensitising the mothers [13]. This package was tested and evaluation revealed the underprivileged women benefitted from this upgraded pregnancy care [13]. This paper aims to understand the role of the skilled healthcare providers in reducing inequity in the utilisation of skilled healthcare services during pregnancy, childbirth, and the postnatal period.

## Materials and methods

### Study design and settings

**Fig. 1:**
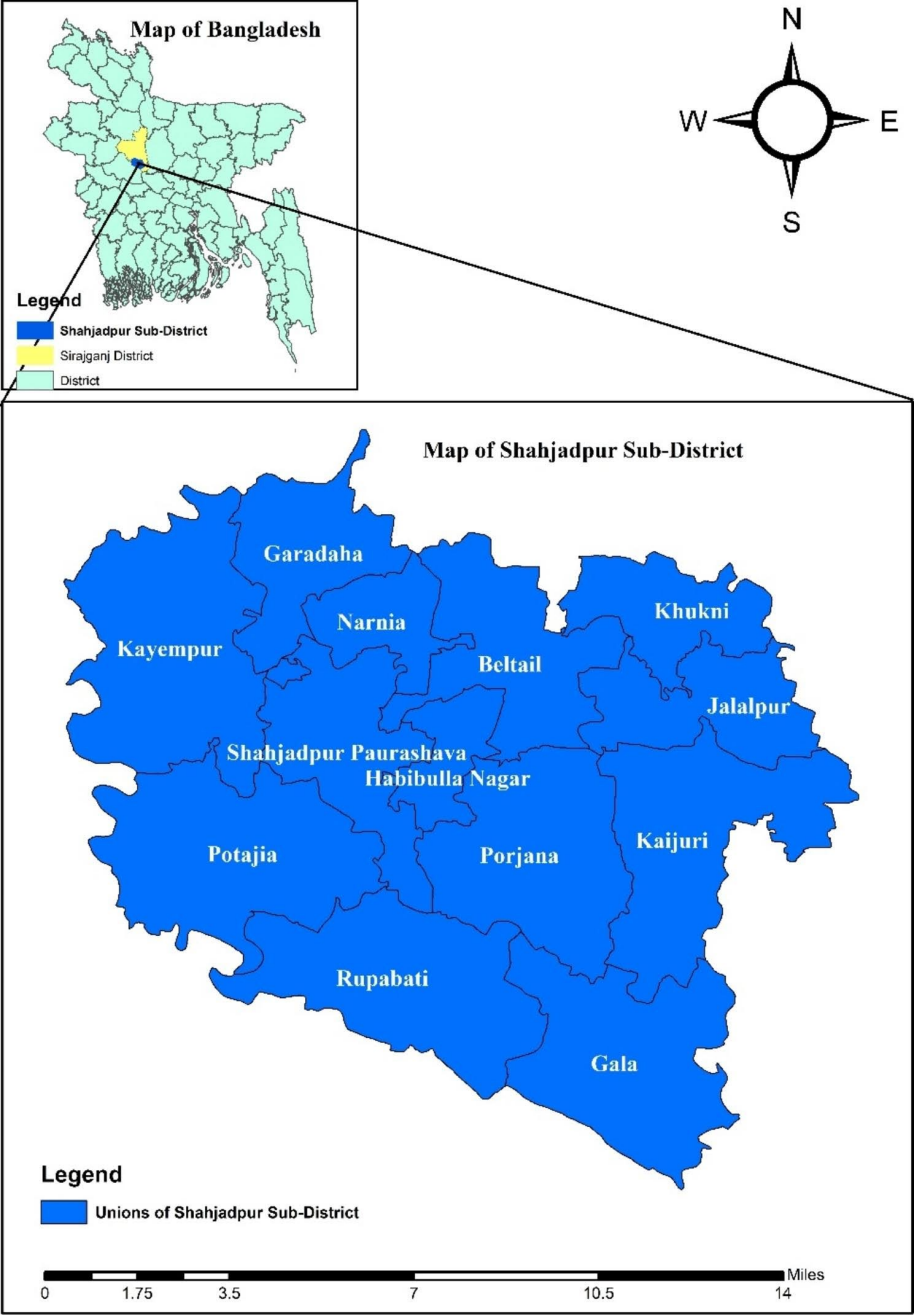
Map of Shahjadpur Sub-District with Unions

The study was quasi-experimental in design. The integrated intervention package was introduced in Shahjadpur, a sub-district of Sirajganj district under the Rajshahi division, Bangladesh (Fig. 1) covering about 600,000 populations, between 2009 and 2012. The baseline and endline surveys were conducted to evaluate the effect of the intervention.

### Sampling and study participants

**Fig. 2:**
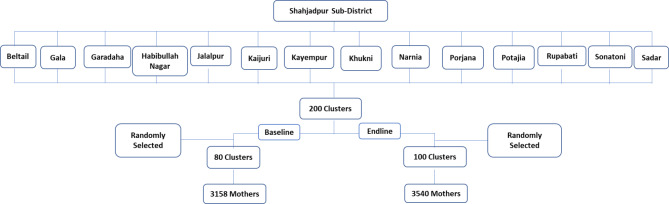
Enrolment process of the study participants

One municipality area and thirteen unions of Shahjadpur were divided into clusters. Each cluster accommodated approximately 3,000 people. Two hundred clusters were then made out of a total population of 583,350. At baseline, 80 clusters (3,158 mothers were interviewed; 28–54 mothers/cluster), and, at endline, 100 clusters (3,540 mothers were interviewed; 23–42 mothers/cluster) were randomly selected (Fig. 2). The data collection methodology and survey questionnaire were the same at both timepoints. Sample size estimation was based on the reduced neonatal mortality (from 37 to 21 per 1,000 live births after 2-years of completion of the intervention) with 95.0% confidence interval and 80.0% power, a minimum of 1,250 mothers who experienced live births were estimated for each survey. A total of 1,290 mothers were required to conduct analysis for the stillbirth outcome also (assuming 30/1,000 births). After taking into consideration a non-response rate of 5%, a total of 1,360 mothers who had an experience of childbirth after 28 weeks of gestation were required. Finally, a total of at least 2,720 recently delivered mothers (delivery occurred in the last 6-months) were estimated in each baseline and endline survey using the design-effect of 2 for cluster sampling. Married women of reproductive age (15–49 years) who had a delivery outcome in the last 6-months before the date of interviews were eligible to participate. The exclusion criteria for the study participants was the mothers who were not permanent residents of the area.

### Data collection

The baseline and endline surveys were conducted in 2009 and 2012 respectively. The survey questionnaire included possession of household assets and demographic characteristics of the mothers, including age, education, care-seeking patterns for antenatal/natal/postnatal periods, latest birth outcomes, newborn care, and neonatal care. The questionnaire was guided by the compendium of Maternal and Newborn Health Tools developed by MEASURE Evaluation [14]. The pre-study questionnaire was written in English, and two bilingual researchers translated it into the local language (Bengali). Another independent bilingual expert back- translated the questionnaire to check for consistencies and to prevent any bias. The final questionnaire was shared in the local language (Bengali). The survey questionnaire was pilot-tested with a small group to ensure its consistency, clarity, and sequencing and to avoid any unnecessary/repeated questions. The questions were finalised after adjusting the contextual thematic aspects for Bangladesh that emerged from the pre-testing on completion of the data collectors training. The trained data collection team comprised of four field research supervisors (FRSs) and 16 Field Research Assistants (FRAs). The FRAs were responsible for the structured interview at the household level and the interview quality was ensured by close monitoring by the FRSs. All the eligible women in each cluster were identified and approached for the interview by a door-to-door visits.

### Data management and quality assurance

An efficient research team led by an experienced supervisor was involved in the data collection. To maintain the quality of data, the team leader supervised the data collection team and was responsible for spot verification of the completeness of every interview. Furthermore, a research investigator (RI) and a project research physician (PRP) were appointed to coordinate the data collection team and checking the data quality on a daily and weekly basis. To ensure the accuracy and data validity, the PRP and RI conducted a significant number of re-interviews for any discrepancies. Simultaneously, an expert programmer team from the Maternal and Child Health Division (MCHD) of icddr,b designed a database template using Dot net (Version-10) software to enter all the data online. The data template housed an advanced design to avoid missing variables and data entry mistakes. The expert data management team entered all the pre- and post-coded data through an online database simultaneously. For post-coding of data, the data management team cooperated with the research team.

### Ethics

This study did not possess more than minimal risk for the study participants. All the study participants were required to give written informed consent before the interviews were conducted. The research team sought approval from the Institutional Review Board (IRB) of icddr,b before the data collection in the field. Since the study only enrolled married women, consent was acquired from the husband on behalf of women under the age of 18.

### Data analysis

Data analysis was done using STATA 13.1 (Stata, College Station, TX, USA). Chi-square test of independence was used to test the crude association between the maternal health indicators and the surveys (baseline and endline) by the socioeconomic status of the respondents. Further, principal component analysis was done to measure respondents’ socioeconomic status by a wealth index derived from the ownership of household assets [15]. The assets included housing materials (e.g. types of roof), housing facilities (e.g. sources of drinking water, types of toilet), durable consumption goods (e.g. television, bicycle, watch, table, chair) and land ownership. Wealth index was then categorised into five ordinal categories: poor, less poor, middle, upper middle, and rich. The concentration indices for inequity measurement were calculated for utilisation of each maternal health care indicators and the respondent’s wealth score. The indicators and scores were then plotted to generate the concentration curves to observe any changes in inequity between the baseline and the endline time period.

### The operational definition of concentration curves and index

The concentration curve delineates inequity by plotting the cumulative percentage of health service utilisation with respect to the cumulative percentage of the population ranked from the poor to the rich. When the concentration curve is conformed to the line of equity at 45°, it then shows the perfect equity. A curve that lies above the perfect equity line means the health service utilisation is more concentrated among the poor, and vice versa. The concentration index gives the magnitude of inequity which is ranged from − 1 to + 1 and is also defined as twice the area between the concentration curve and the line of equity. Perfect equity is achieved when the index value is zero; the index value closer to -1 means the disproportionate concentration of health service utilisation increases among the poor whereas the disproportionate concentration of health service utilisation increases among the rich if the index value gets closer to + 1 [16].

Maternal healthcare-seeking indicators included in this paper were ANC and PNC by a skilled provider and delivery by a skilled birth attendant (SBA). These indicators are binary variables, creating a problem with the standard concentration index which is not necessarily always within the range of -1 to + 1 [17]. Wagstaff developed a modified concentration index by re-scaling the standard index to keep unscathed the relative inequity variance property of the concentration index [18]. For the corrected concentration index for this study, *conindex* command of STATA has been used [19].

## Results

Table 1 describes demographic characteristics of the mothers during the baseline and endline survey periods. There were no significant differences across the five categories of the wealth index where the mothers belonged. One-fifth of the mothers in both the baseline and the endline surveys were aged below 20 years across the five wealth index categories. At baseline, mothers in richer quintiles were significantly more likely to have higher educational level, but no significant differences were found among the groups at the endline period. A similar significant trend was seen between wealth index and husband’s educational level during both the baseline and the endline surveys. Almost all the mothers were homemakers and two-thirds of the mothers had experienced more than one pregnancy.

**Table 1:** Sociodemographic characteristics of recently delivered mothers

Sociodemographic characteristics	Baseline (% of mothers) N = 3158					Endline (% of mothers) N = 3540				
	Poor	Less poor	Middle	Upper middle	Rich	Poor	Less poor	Middle	Upper middle	Rich
	n = 631	n = 635	n = 629	n = 632	n = 631	n = 714	n = 707	n = 703	n = 706	n = 710
Maternal age (in years)										
≤ 19	20.8	22.7	22.6	23.4	23.8	19.9	22.4	19.6	22.5	20.1
20–29	65.0	61.7	60.1	62.8	63.2	60.9	58.7	62.0	59.8	61.3
≥ 30	14.3	15.6	17.3	13.8	13.0	19.2	19.0	18.4	17.7	18.6
*p-value*	0.454					0.858				
Maternal education level (in years)										
No schooling	46.1	40.0	35.5	30.5	28.1	33.6	28.9	32.2	30.7	31.6
1–5	30.1	32.4	34.3	34.7	34.2	37.5	36.8	32.7	36.2	35.8
6–7	14.6	15.9	18.9	21.2	20.1	18.9	21.6	20.2	19.5	18.1
≥ 8	9.2	11.7	11.3	13.6	17.6	9.9	12.7	14.9	13.6	14.5
*p-value*	0.000					0.161				
Husband education level (in years)										
No schooling	52.5	47.6	41.5	38.0	34.2	42.0	43.0	42.5	42.2	40.9
1–5	24.1	22.1	27.8	25.5	27.3	28.9	24.9	25.3	26.1	27.4
6–7	8.4	11.0	11.8	13.3	11.4	12.5	13.6	12.7	10.4	8.9
≥ 8	15.1	19.4	18.9	23.3	27.1	16.7	18.5	19.5	21.3	22.9
*p-value*	0.000					0.059				
Occupation										
Homemaker	94.0	95.1	94.6	95.4	94.5	93.0	91.0	91.5	91.2	92.5
Others	6.0	4.9	5.4	4.6	5.6	6.7	8.8	8.4	8.4	7.5
*p-value*	0.808					0.648				
Reproductive History										
Primi-para	32.3	32.0	31.3	39.4	37.6	27.5	33.1	30.3	32.0	33.1
Multi-para	67.7	68.0	68.7	60.6	62.4	72.6	66.9	69.7	68.0	66.9
p-value	0.000					0.110				

Table 2 draws a comparison between the baseline and endline estimates of antenatal, delivery, and postnatal care services with skilled healthcare providers, showing significant increases among the mothers in all five wealth quintiles. At the baseline survey period, 6% and 14% of the mothers in the poor and rich groups received more than four ANC services from the skilled providers respectively and at the endline period, more than one-third of the mothers received the ANC services across each of the wealth index groups. Similar significant differences were found for using skilled birth attendants between the baseline and the endline periods across all socioeconomic groups. The SBA usage increased by over 20% in the poor, less poor, and middle quintiles. The change was smaller but remained significant in upper middle and rich quintiles. Use of skilled PNC services was extremely low before the intervention in all socioeconomic groups (2.4-5.9%) and after the intervention, there was a significant improvement in all quintiles by around 20%. The inequity in the utilisation of more than four skilled ANC services, skilled childbirth, and skilled PNC services declined significantly over the intervention period. The reduction in the rich-poor ratio was largest in the utilisation of 4+ skilled ANC services which reduced from 2.4:1 to 1.1:1, and in PNC services from 2.5:1 to 1.0:1 between the baseline and the endline periods.

**Table 2:** Maternal healthcare seeking behavior by their socioeconomic status during the baseline and the endline

MNH care services	% of mothers					
	Poor/	Less poor/	Middle/	Upper middle/	Rich/	Rich-Poor ratio
	Poorest	Poorer	Poor	Less poor	Least poor	
4+ANC by skilled providers						
Baseline	5.9	6.6	7.6	10.8	14.0	2.4:1
Endline	31.5	34.9	30.6	30.9	35.5	1.1:1
*p-value*	0.000	0.000	0.000	0.000	0.000	
Delivery by skilled providers						
Baseline	20.6	21.1	26.4	29.0	35.3	1.7:1
Endline	45.0	47.1	45.7	45.2	43.7	1.0:1
*p-value*	0.000	0.000	0.000	0.000	0.002	
PNC by skilled providers						
Baseline	2.4	3.0	3.3	3.0	5.9	2.5:1
Endline	24.9	24.9	27.5	23.7	25.9	1.0:1
*p-value*	0.000	0.000	0.000	0.000	0.000	

**Fig. 3:**
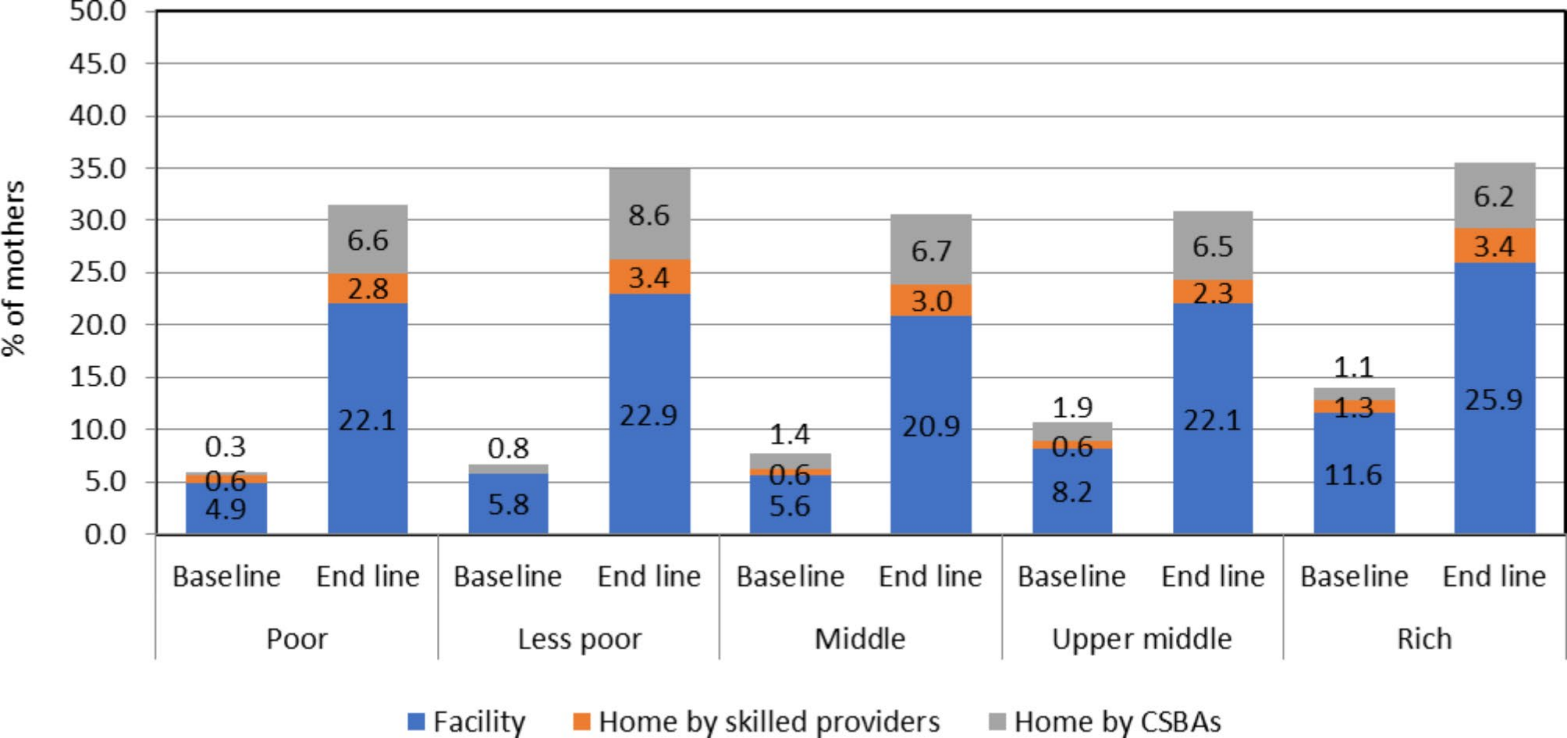
Source of skilled 4+ ANC by socioeconomic status during the baseline and endline survey

Figure 3 shows the proportion of mothers receiving 4+ skilled ANC services increased during the study period across the five wealth index groups. A four-fold increase was observed among the poorer quintiles receiving skilled ANC services at the facilities during the endline period compared to the baseline period. The proportion also significantly increased for CSBAs providing ANC services at home. Despite this, by baseline, more than one-fifth opted for ANC services at the facility compared to around 3% and 6% receiving ANC at home by skilled providers and CSBAs respectively.

**Fig. 4:**
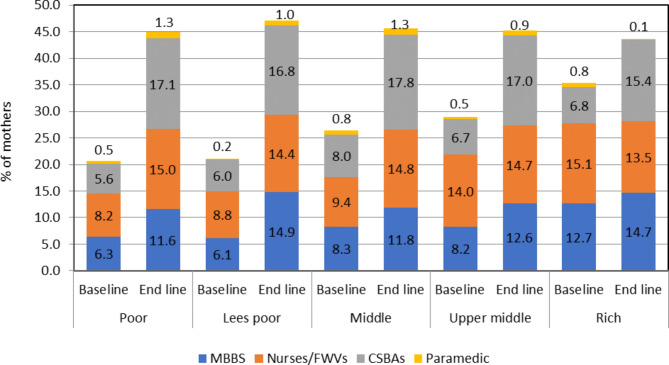
Source of skilled delivery by socioeconomic status during the baseline and endline survey

Figure 4 demonstrates mothers from all wealth index groups were more likely to have CSBAs at delivery compared to either doctors or nurses/FWVs at the endline period.

**Fig. 5:**
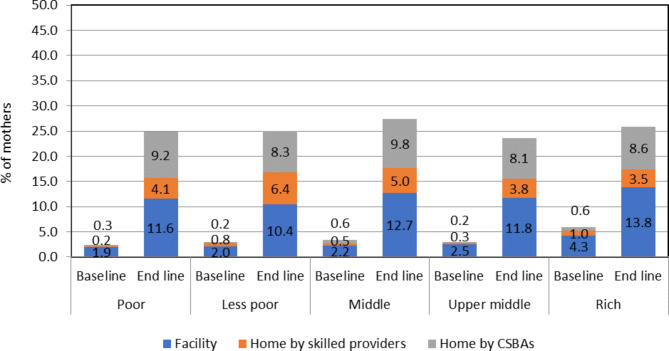
Source of skilled PNC by socioeconomic status during the baseline and endline survey

Figure 5 indicates at baseline the utilisation of the facility and CSBAs for PNC services was noteworthy in all the wealth index groups also.

**Fig. 6:**
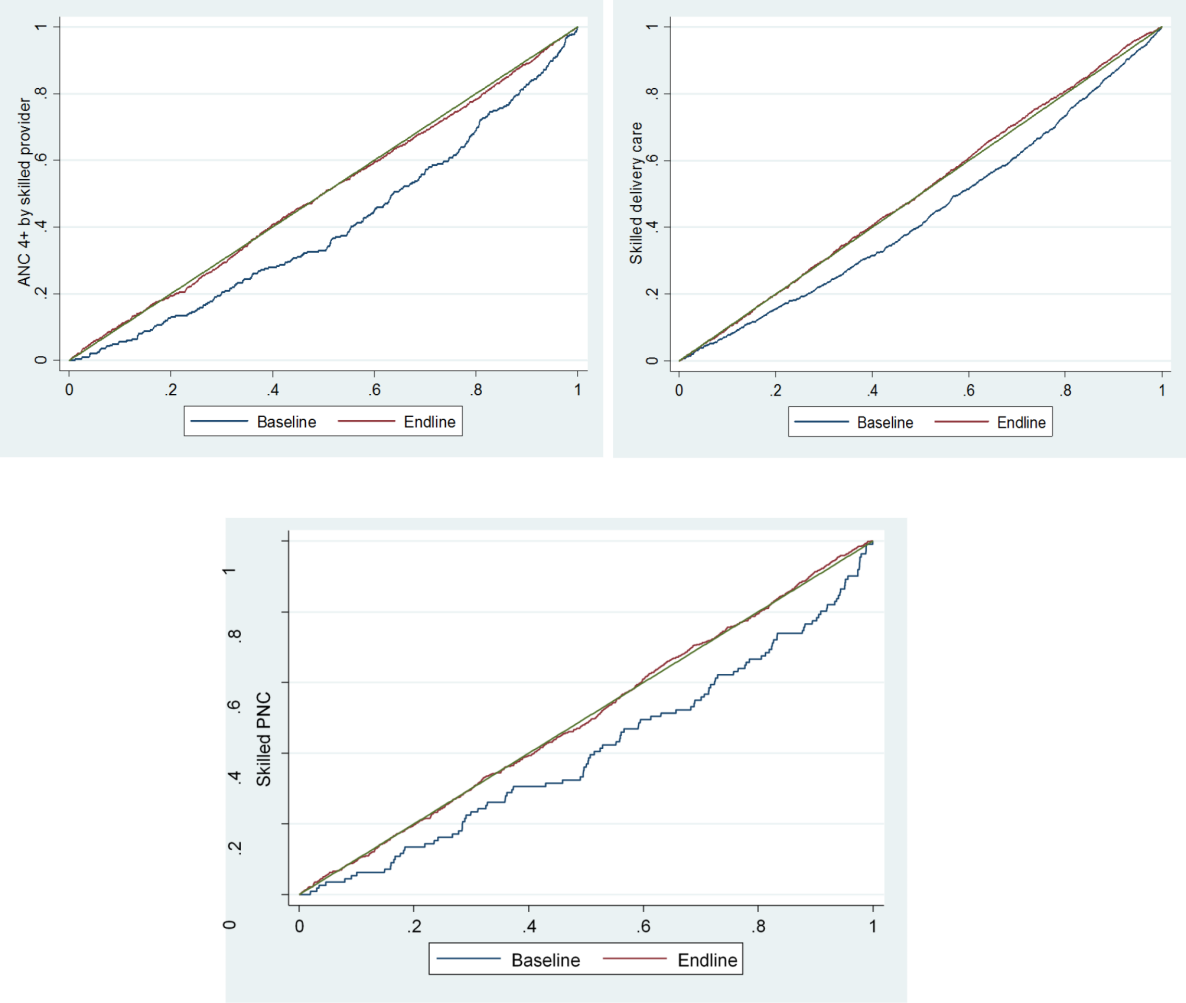
Concentration curve of skilled 4+ ANC, skilled delivery, and skilled PNC utilisation by study period

Figure 6 shows the equity gap reduction between poor and rich after the intervention for skilled ANC, childbirth, and PNC services in comparison to the baseline.

Table 3 shows the concentration index decreased from 0.220 to 0.013 (p < 0.001) for 4+ skilled ANC utilisation. The rich-poor ratio for skilled childbirth reduced from 1.7:1 to 1.0:1 and the concentration index also declined from 0.161 to -0.021 (p < 0.001). A similar reduction was also observed in the utilisation of skilled PNC; where the rich-poor gap decreased from 2.5:1 to 1.0:1 and the related concentration index declined from 0.197 to -0.004 (p < 0.001). Figure 6 shows the concentration curves for the utilisation of 4+ skilled ANC, childbirth, and PNC between the baseline and the endline periods.

**Table 3:** Measure of inequity (Concentration Index and 95% Confidence Interval) by study period

MNH care services	Baseline			Endline			% Change	p-value
	CI	LL	UL	CI	LL	UL		
4+ ANC by skilled providers	0.220	0.219	0.221	0.013	0.0125	0.0139	-0.207	< 0.001
Delivery by skilled providers	0.161	0.159	0.161	-0.021	-0.021	-0.020	-0.181	< 0.001
PNC by skilled providers	0.197	0.177	0.215	-0.004	-0.012	0.003	-0.201	< 0.001

CI = Concentration Index

LL = Lower Limit

UL = Upper Limit

## Discussion

This study demonstrates the inequity prevalent between poor and rich communities in accessing essential maternal healthcare services, including ANC, delivery, and PNC. However, after the intervention, the inequity gap reduced and mothers received improved healthcare services with the presence of skilled providers. Maternal healthcare services during the antepartum, intrapartum, and postpartum periods were better utilised by the mothers in all wealth index groups, contributing to the favorable results of the intervention. Other demographic factors, such as level of education, maternal occupation status and number of pregnancies, played a minimal role over the utilisation of skilled care across all the wealth groups.

Different maternal healthcare services have been provided through district hospitals, upazila health complexes, union health and family welfare centers, and community clinics, since the 1980s by the Government of Bangladesh (GoB) [20]. But the service delivery module did not guide to reach the poor. This recently changed through the attempt at ensuring universal health coverage for all [21]. This study was proposed based on the inequity found in the MNCH care in Bangladesh from 2007 and persisting until 2017. The Bangladesh Demographic and Health Surveys reported that a large proportion of pregnant women in the poorest wealth quintiles doesn’t receive ANC from a medically trained provider and while almost all the pregnant women in the richest quintile received ANC [8, 22–24]. Further studies suggest half of the pregnant women in Bangladesh give birth at home and the majority is conducted by unskilled birth attendants. Furthermore, references discussed in background of this study talks for the long drawn inequity among the wealth index groups on the health facility delivery and uptake of PNC (8). These inequities in maternal healthcare services were observed to decrease from 2007 to 2017-18 but large gaps still exist in Bangladesh. Hence, specific actions for the government includes initiation of actions to provide universal health coverage of essential interventions and packages of service delivery prioritising availability of skilled healthcare providers is emphasised.

Connecting obstetric providers to the women in poor socioeconomic groups impacted by increasing facility maternity care [25]. This study drew attention to the impact of the intervention on reducing rich-poor inequalities in access to CSBAs and skilled healthcare providers despite wealth index and their respective physical remoteness, where scarcity of the skilled healthcare providers has previously been an obstacle for the basic and comprehensive emergency obstetric care. We found that the integrated intervention facilitates significant progress in skilled maternal healthcare utilisation in the selected sub-district of Bangladesh. All the key indicators of the utilisation of skilled maternal healthcare improved in this area over the 4-years implementation of the intervention package. Skilled ANC increased several fold, skilled delivery care doubled among the poor groups and similarly, a robust improvement in skilled PNC was observed among the poor women. The integrated intervention package focusing on coverage of skilled healthcare providers like CSBAs is assumed to provide opportunities for women to be informed on the necessity of the facility care as well. Another article generated from this very study also showed positive effects on the skilled provider’s care during pregnancy and delivery in more details in remote areas by poor mothers [13]. A longitudinal multi-country study that was conducted during 2001–2011 in India, Nepal, and Bangladesh also echoed that women from the rich wealth quintile had an upper hand in utilising the facility delivery care in all the countries [26].

The ANC is rationalised to maintain the continuum of care [27], for example, facilitation of mother’s access to skilled delivery and postpartum care [28]. One of the important findings of this study was a decreased rich-poor gap for the skilled ANC services at the end of the intervention. The intervention was successful in improving equitable access to the ANC services that reflected in the smaller gaps between rich and poor for availing 4+ ANC from skilled providers. Provision of skilled ANC is expected to encourage women, especially the poor, to seek skilled care during and after delivery (PNC). This might prevent the practice of seeking care too late, at the height of a maternal emergency, thus saving money for advanced management as well as reducing maternal mortality. Ultimately, these implications would end-up with a cost-effective benefit of improved maternity services on mortality and morbidity reduction [27]. Despite the reduction of the rich-poor gap and the significant improvement in 4+ ANC by the poor, the 4+ ANC rate from the skilled healthcare providers remained considerably low among the mothers (around one-third) in the sub-district at endline. Therefore, greater efforts are needed in obtaining better access to skilled ANC services for all mothers. However, the observed significant increase in the utilisation of the facility for ANC services at the endline would be conclusive about the positive effect of the intervention.

Although significant changes in using skilled delivery care had been marked between the baseline and the endline across the five wealth index categories, overall utilisation of skilled birth attendants was doubled at the end of the intervention among the poor women. This contributed to the equalised rich-poor gap by the endline. CSBAs proportionate coverage by the intervention among all the quintiles might result in reducing inequity gaps. Facility delivery among poorer women was also doubled after the intervention. This is likely due to the intervention raising awareness of services, through counseling by CSBAs and CSGs. That stimulated an efficient follow-up of a fundamental second option of facility delivery for more effective intrapartum strategies [29]. It is encouraged to conduct delivery at facility, especially for high-risk women; because home delivery, even with a skilled birth attendant, has more complex logistic issues. For example, recognition of initial immediate management of complications and cost-effective transport mechanism for the compliance to their referral decision [30].

In Bangladesh, facility delivery remains low despite the improved healthcare infrastructure [29, 30]. Home-based intrapartum care could be advocated to reduce the burden of recognition of complications and arrangement of transport on families. Facility maternity care placed many countries with maternal mortality less than 200 deaths or even lower per 100,000 live births [29]. Nevertheless, facility-based care alone is not enough to reduce maternal mortality [29]. The MIRA study in Makwanpur, Nepal, and another similar study in Kenya concluded that simply strengthening health facilities is unlikely to influence maternal and perinatal care-seeking practices in a situation of preference to community-based management and close access to the facility [26, 28, 31].

Reduction in the risk of adverse maternal and neonatal outcomes is also associated with PNC timing [29]. After the intervention, skilled PNC increased several folds and a significant decrease in the rich-poor gap was observed. Despite the improvement, overall PNC coverage remained low, at one-fourth in all wealth index groups at the endline, indicating a further emphasis is required to increase PNC utilisation. Generally, PNC within 2 days following childbirth is very high for facility delivery in comparison to home delivery (8). Association between the facility delivery and the PNC was not measured in this study but descriptive statistics revealed that the facility-based PNC followed after the similar trend of facility delivery among the poor. However, a study that used Indian National DHS survey 2007-08 reported many factors including socioeconomic background is a significant determinant of the PNC utilisation scope and mothers and babies born to wealthy family utilise the service more than the mothers and babies of the below strata [32]. As already mentioned this intervention did not take into full account the resources needed to achieve high uptake of skilled PNC at the facility after childbirth. Therefore, change in preference from home to facility delivery also has prospects to increase the optimal skilled PNC among the poor mothers.

### Strengths and Limitations

This intervention is the first of its kind combining the most essential maternal healthcare service components together as an integrated package to maximise the service quality related to maternal health in a remote setting like ours. Further, this intervention successfully employed one skilled provider per ten thousand population in community setting as per World Health Organization (WHO) health human resource guidelines, ensured community engagement and the overall efforts translated into the improved maternal healthcare service utilisation by the endline. However, this study did not evaluate cost-benefit, thus preventing the detection of its sustainability. This experimental study also observed the effect size based on a before-and-after design without comparing it with a control group. Therefore, it is difficult to exclude the possibility of other intervention effects. This study did not account for any other health system-related factors other than skilled birth provider which was associated with inequity. In addition to that, recall bias might have affected the quality of household data. However, the inclusion of women who had delivered in the last 6 months helped to minimise these errors. The study used a quasi-experimental approach to assess the effect of the implementation of the intervention rather than a randomised controlled trial.

## Conclusion

The significant improvements in skilled ANC, delivery care, and PNC by the mothers in poorer wealth quintiles in this study was likely influenced by the implementation of the integrated maternal healthcare intervention. Since the proportion of mothers using all skilled maternity care services remained low in this study, that greater progress is needed for rural women. Further research should focus on the community demands of health system issues to overcome barriers to achieve progress. Despite this, the study results indicate that the existing wealth inequity in the health systems can be reduced by adopting the intervention approaches, involving both the community and the health facilities. In this intervention, establishing community support groups created community awareness that might support poor pregnant women to reach health facilities either for routine pre- and post-natal care or during delivery emergencies. This study also indicated that the intervention successfully equipped and boosted the skilled health workforce through refresher training on MNCH to ensure the quality of care to the mothers and their infants.

## Data Availability

Anonymised participant data will be shared by the corresponding author after corresponding author’s institutional approval, following a reasonable submitted request.

## Abbreviations

AMTSL: Active management of third stage labor
ANC: Antenatal care
AusAID: Australian Agency for International Development
BDHS: Bangladesh Demographic and Health Survey
CSBAs: Community skilled birth attendants
CSGs: Community support groups
EmOC: Emergency Obstetrical Care Services
FRAs: Field Research Assistants
FRSs: Field research supervisors
GoB: Government of Bangladesh
icddr,b: International Centre of Diarrheal Disease Research, Bangladesh
IRB: Institutional Review Board
MCHD: Maternal and Child Health Division
MHVS: Maternal Health Voucher Scheme
MNCH: Maternal, Neonatal, and Child Health
MNH: Maternal and neonatal health
PNC: Postnatal care
PPH: Postpartum hemorrhage
PRP: Project research physician
RI: Research investigator
SBA: Skilled birth attendant
SDG: Sustainable Development Goal
UHC: Universal Health Coverage
WHO: World Health Organization
